# A Laboratory-Based Evaluation of Four Rapid Point-of-Care Tests for Syphilis

**DOI:** 10.1371/journal.pone.0091504

**Published:** 2014-03-11

**Authors:** Louise M. Causer, John M. Kaldor, Christopher K. Fairley, Basil Donovan, Theo Karapanagiotidis, David E. Leslie, Peter W. Robertson, Anna M. McNulty, David Anderson, Handan Wand, Damian P. Conway, Ian Denham, Claire Ryan, Rebecca J. Guy

**Affiliations:** 1 Kirby Institute, University of New South Wales, Sydney, Australia; 2 School of Population and Global Health, University of Melbourne, Carlton, Australia; 3 Victorian Infectious Diseases Reference Laboratory, Melbourne, Australia; 4 SEALS Area Serology Laboratory, Prince of Wales Hospital, Randwick, Australia; 5 Sydney Sexual Health Centre, Sydney, Australia; 6 Burnet Institute, Melbourne, Australia; 7 Melbourne Sexual Health Centre, Carlton, Australia; California Department of Public Health, United States of America

## Abstract

**Background:**

Syphilis point-of-care tests may reduce morbidity and ongoing transmission by increasing the proportion of people rapidly treated. Syphilis stage and co-infection with HIV may influence test performance. We evaluated four commercially available syphilis point-of-care devices in a head-to-head comparison using sera from laboratories in Australia.

**Methods:**

Point-of-care tests were evaluated using sera stored at Sydney and Melbourne laboratories. Sensitivity and specificity were calculated by standard methods, comparing point-of-care results to treponemal immunoassay (IA) reference test results. Additional analyses by clinical syphilis stage, HIV status, and non-treponemal antibody titre were performed. Non-overlapping 95% confidence intervals (CI) were considered statistically significant differences in estimates.

**Results:**

In total 1203 specimens were tested (736 IA-reactive, 467 IA-nonreactive). Point-of-care test sensitivities were: Determine 97.3%(95%CI:95.8–98.3), Onsite 92.5%(90.3–94.3), DPP 89.8%(87.3–91.9) and Bioline 87.8%(85.1–90.0). Specificities were: Determine 96.4%(94.1–97.8), Onsite 92.5%(90.3–94.3), DPP 98.3%(96.5–99.2), and Bioline 98.5%(96.8–99.3). Sensitivity of the Determine test was 100% for primary and 100% for secondary syphilis. The three other tests had reduced sensitivity among primary (80.4–90.2%) compared to secondary syphilis (94.3–98.6%). No significant differences in sensitivity were observed by HIV status. Test sensitivities were significantly higher among high-RPR titre (RPR≥8) (range: 94.6–99.5%) than RPR non-reactive infections (range: 76.3–92.9%).

**Conclusions:**

The Determine test had the highest sensitivity overall. All tests were most sensitive among high-RPR titre infections. Point-of-care tests have a role in syphilis control programs however in developed countries with established laboratory infrastructures, the lower sensitivities of some tests observed in primary syphilis suggest these would need to be supplemented with additional tests among populations where syphilis incidence is high to avoid missing early syphilis cases.

## Introduction

Timely diagnosis and treatment of syphilis is crucial to reduce morbidity, and onward transmission of syphilis to sexual partners and newborns, in whom consequences can be devastating [1]. Congenital syphilis is almost entirely preventable with early identification of maternal infection followed by prompt treatment [2]. Syphilis can also increase susceptibility to acquiring HIV infection and increase transmissibility [3], [4]. In resource limited settings with poor access to laboratories or syphilis screening, rapid point-of-care (POC) tests have the potential to increase numbers of people tested, and provide clinical services with the opportunity to offer treatment immediately during the same consultation.

Syphilis testing is recommended in clinical guidelines as part of antenatal screening, for people at high risk, for those with symptoms and following sexual contact with confirmed cases [5]–[7]. There has been substantial enthusiasm and support for the implementation of POC tests for syphilis in resource limited settings [8]–[10], with several countries having recently adopted POC tests into their national antenatal screening policies [11]. This approach is supported by evidence from laboratory and field evaluations of syphilis POC tests which demonstrate good performance, acceptability and cost effectiveness [12]–[18], however very little has been published on the performance of these tests during different clinical stages of infection and among those with HIV co-infection which may influence the host response to syphilis [19].

This paper presents results of a large dual-site, laboratory-based, head-to-head evaluation of performance of four commercially available rapid POC syphilis tests using archived sera that includes analysis by syphilis stage, HIV status and non-treponemal antibody titre.

## Methods

### Ethical approval

Ethical approval was granted by the South East Sydney and Illawarra Area Health Service Human Research Ethics Committee (HREC), Melbourne Health HREC and University of New South Wales HREC. Consent was not required from individuals as it was a retrospective study of de-identified stored samples that has previously been tested with the goal standard syphilis assays. Linked clinical data were anonymized and de-identified prior to analysis.

### Setting

Rates of syphilis in Australia are highest among two distinct populations - gay men living in urban centres and Aboriginal and Torres Strait Islander people living in remote regions across Australia [20]. The incidence of syphilis in gay men with HIV infection is five-times higher than the incidence in MSM without HIV (2.5 vs. 0.5 per 100 person years) [21]. Study sera were selected from two major laboratories in Australia, both conducting a high volume of syphilis testing. These laboratories also are the primary pathology providers for two large, urban sexual health clinics in Sydney and Melbourne from which corresponding patient demographic and clinical information was extracted. These clinics have extensive clinical expertise in STI diagnosis and management and provide sexual health services to a significant proportion of the gay population in these cities.

### Study design

We assessed the performance of four syphilis POC tests by comparing results to routinely performed reference treponemal tests (immunoassays [IA]) conducted at the laboratories. Linked clinical data for a subset of specimens were extracted from patient medical records. Inter-technician variability and operational characteristics of the tests were also assessed.

### Syphilis POC tests

POC tests were selected to include commercially available tests with traditional as well as novel test platforms: (i) Determine Syphilis TP® (Inverness Medical Japan Co, Ltd, Chiba, Japan); (ii) Onsite Syphilis Ab Combo Rapid Test® (CTK Biotech, San Diego, CA, USA); (iii) SD Bioline Syphilis 3.0® (Standard Diagnostics Inc, Kyonggi-do, Korea); and (iv) DPP Syphilis Screen and Confirm Assay® (Chembio Diagnostic Systems Inc, Bedford, NY, USA). All four tests use an immunochromatographic strip design with the appearance of a visible coloured line if treponemal antibodies are detected in the specimen. The DPP Syphilis test, in addition, simultaneously detects non-treponemal antibodies by a separate coloured line on the same test strip.

### Laboratories and serological reference tests

The respective reference treponemal immunoassay (IA) tests used by Victorian Infectious Diseases Reference Laboratory (VIDRL) in Melbourne and South Eastern Area Laboratory Service (SEALS) in Sydney are: (i) Trepanostika TP recombinant enzyme immunoassay® (bioMerieux, Boxtel, The Netherlands) [22] and (ii) Architect Syphilis TP chemiluminescence assay® (Abbott, Wiesbaden, Germany) [23], [24]. Both laboratories perform quantitative rapid plasma reagin (RPR) tests using BD Macro – Vue RPR Card Tests® (Becton, Dickinson and Co, MD, USA). In Australia, a “reverse” screening algorithm is used, i.e. treponemal IA followed by a quantitative RPR for IA reactive specimens. Both laboratories participate in an ongoing external quality assurance program (Royal College of Pathologists of Australia Quality Assurance Program).

Reference test results were extracted from the laboratory databases or patient medical records for comparison with POC test results. A specimen from a patient with a previously documented reactive treponemal IA result was not retested and was considered to be IA reactive for the study analyses.

### Specimen selection and categorisation

Specimens were selected to include approximately equal numbers of IA reactive and IA non-reactive samples, and a range of RPR titres.

#### a. Serological categorisation

Specimens were categorised by treponemal IA and by non-treponemal (RPR) reference test results as *high RPR titre syphilis* (IA reactive, RPR≥8); *low RPR titre syphilis* (IA reactive, RPR = 1, 2 or 4); *inactive* syphilis (IA reactive, RPR non-reactive); *no syphilis* (IA and RPR non-reactive); and *biological false positives* (IA non-reactive, RPR ≥2).

#### b. Clinical categorisation

Demographic (age, gender, gender of sexual partners in last 12 months) and clinical data (HIV status, CD4 count, syphilis stage) were extracted from medical records. Specimens were categorised by clinical stage according to the definitions in Table 1.

**Table 1:** Clinical Syphilis Case Definitions.

**Primary syphilis** – primary chancre with reactive syphilis serology and/or PCR and/or dark ground positive test(s)
**Secondary syphilis** – systemic symptoms typical of syphilis with reactive syphilis serology, plus mucocutaneous lesions (which may be dark field microscopy and/or PCR positive)
**Early latent syphilis** – asymptomatic with reactive syphilis serology (on two occasions) and non-reactive serology results within the last 2 years or, if not previously tested, likely time of infection in the last 2 years
**Late or unknown duration latent syphilis** – asymptomatic with reactive syphilis serology and no prior testing or prior testing >2 years since the last test
**Past treated syphilis** – documented adequate treatment for syphilis and has achieved adequate serological and clinical response on follow up

Positive syphilis serology – either reactive immunoassay ± reactive RPR (need two sets of positive results to confirm if asymptomatic if no prior history of syphilis or reactive RPR with a fourfold titre increase if past treated syphilis).

### POC testing

Technicians performing the POC tests completed training to ensure consistency in the conduct and interpretation of results. Specimens were tested according to the manufacturer's instructions by one study technician blinded to the reference results. The POC test results were interpreted and recorded on a laboratory data record sheet. A second blinded study technician, independently interpreted and recorded the POC test result within 1 minute of the first read. Discrepancies were discussed and a consensus reached and recorded. Reasons for discrepancies were noted. Invalid tests were repeated using a new test and recorded as such.

### Operational characteristics

We assessed selected operational characteristics of each test by self-administered questionnaire among the technicians performing the POC tests. Questions focused on ease of test use and interpretation of the results.

### Data analysis

The sensitivity and specificity for each syphilis POC test compared to the treponemal IA reference test results was calculated by standard methods and a kappa (k) value was calculated for each test as a summation of the overall performance using Stata (StataCorp. Release 12. College Station, TX). Results were stratified by HIV status and RPR titre (among IA reactive specimens). POC test sensitivity was also compared to clinical stage of syphilis. For the analyses presented here, DPP POC test sensitivity refers the treponemal line result compared to reference IA to allow for consistent comparison across all four POC tests.

To compare performance between POC tests and between subcategories, 95% confidence intervals (CI) were calculated [25] for each estimate (sensitivity and specificity). Statistical significance of a difference in estimates was based on non-overlapping CI. P-values were calculated using a Chi-squared test for overall test sensitivities only.

Inter-observer variability to determine the discrepancy rate between the two technicians recording results was calculated as the number of test results which differ between the two technicians x 100/total number of tests performed using the same serum specimens.

## Results

### Sample characteristics

In total, 1203 specimens (678 from Melbourne and 525 from Sydney) were tested with each of the four syphilis POC tests. The median age of the cases was 35 years (range 18–85) and 83.4% were men. Additional patient demographic and clinical information were available for 878 (73%) specimens. About half (50.9%) of the male cases identified as having a least one same sex partner during the 12 months, prior to specimen collection. One hundred and fifty four (12.8%) cases were HIV positive and 67 (5.6%) had a most recent CD4 count of <500 cells/mm^3^.

### Clinical categorisation

As presented in Table 2, 53 cases (4.4%) had primary syphilis, 70 (5.8%) had secondary syphilis, 91 (5.8%) had early latent syphilis, and 25 (2.1%) had late latent infection or infection of unknown duration; 248 (20.6%) were documented as having past treated syphilis infection; four cases had a clinical diagnosis of syphilis with no stage specified; 387 (32.2%) did not have syphilis nor a history of syphilis infection. Among those with primary syphilis, 64.7% had an RPR titre >1∶8 while among those with secondary syphilis, 92.9% had an RPR>1∶8.

**Table 2:** Selected demographic and clinical characteristics of specimens tested (n = 1203).

Variable	N (%)
Male	1003 (83.4)
Female	200 (16.6)
Median age in years	35
Age range in years	18–85
*Additional clinical Information*	
Available	878 (73.0)
Unavailable	325 (27.0)
*Among men, same sex partner in last 12 months*	
Yes	612 (50.9)
No	125 (10.4)
Unavailable	466 (38.7)
*HIV status*	
Positive	154 (12.8)
Negative	724 (60.2)
Unavailable	325 (27.0)
*CD4 count (cells/mm^2^) among HIV positive*	
<200	3 (0.2)
200–<500	64 (5.3)
≥500	63 (5.2)
Unknown	24 (2.0)
Unavailable	325 (27.0)
*Clinical syphilis diagnosis*	
Primary	53 (4.4)
Secondary	70 (5.8)
Early latent	91 (7.6)
Late Latent/unknown duration	25 (2.1)
Past treated	248 (20.6)
Syphilis, no stage specified	4 (0.3)
Not syphilis	387 (32.2)
Unavailable	325 (27.0)
*Serological Syphilis Categorisation*	
IA reactive/RPR reactive (R≥8)	404 (33.6)
IA reactive/RPR reactive (R = 1:1, 2 or 4)	121 (10.1)
IA reactive/RPR non-reactive	211 (17.5)
IA non-reactive/RPR reactive (R≥2)	27 (2.2)
IA non-reactive/RPR non-reactive (RPR≤1)	242 (20.1)
IA non-reactive/RPR not done*	198 (16.5)

* Syphilis testing in Australia involves screening with a treponemal antibody test, followed by RPR, if positive, to stage disease. 198 specimens with non-reactive IA did not have routinely performed RPR results available.

### Serological categorisation

Among the 1203 specimens, 736 (61.2%) were reference IA reactive and 467 (38.5%) were non-reactive. RPR results were available for 1,005 (83.5%) specimens and used to further stratify the specimens as described in Table 2. Among the IA reactive specimens, 404 were high RPR titre (RPR≥1∶8) syphilis, 121 were low RPR titre (RPR = 1∶1, 2, or 4) syphilis, and 211 were RPR non-reactive (inactive syphilis). Among the IA non-reactive, 242 were RPR nonreactive or RPR≤1 (not syphilis) and 27 had an RPR ≥1∶2 (biological false positives).

### Sensitivity and specificity

POC sensitivities and specificities overall, stratified by HIV status, CD4 count are shown in Table 3. Determine TP showed the highest sensitivity (97.3%) and the difference was statistically significant to the other three tests (p<0.001). POC test specificities ranged from 96.4–98.5% with no significant differences between tests. The kappa statistic for Determine (0.94, 95% CI: 0.92–0.96) was statistically significantly higher than the other three tests which ranged from 0.84–0.88.

**Table 3:** Syphilis POC test sensitivity and specificity (compared to reference Treponemal immunoassay), overall and by HIV status and CD4 cell count.

			Determine		Onsite		DPP (Trep)		Bioline	
		N	Sens	Spec	Sens	Spec	Sens	Spec	Sens	Spec
		(IA+/IA−)*	(95% CI)	(95% CI)	(95% CI)	(95% CI)	(95% CI)	(95% CI)	(95% CI)	(95% CI)
Overall		1203	97.3	96.4	92.5	97.0	89.8	98.3	87.8	98.5
		(736/467)	(95.8–98.3)	(94.1–97.8)	(90.3–94.3)	(94.9–98.3)	(87.3–91.9)	(96.5–99.2)	(85.1–90.0)	(96.8–99.3)
HIV status	Neg	724	95.9	97.8	91.2	97.2	89.8	98.6	86.5	98.9
		(364/360)	(93.1–97.6)	(95.5–99.0)	(87.7–93.8)	(94.8–98.6)	(86.1–92.7)	(96.6–99.5)	(82.5–89.8)	(97.0–99.6)
	Pos	154	96.9	81.5	94.5	85.2	89.8	88.9	87.4	88.9
		(127/27)	(91.6–99.0)	(61.3–93.0)	(88.6–97.6)	(65.4–95.1)	(82.8–94.2)	(69.7–97.1)	(80.1–92.4)	(69.7–97.1)
CD 4 count	<500	67	94.6	91.7	90.9	100.0	89.1	100.0	81.8	100.0
(cells/mm3)		(55/12)	(84.0–98.6)	(59.8–99.6)	(79.3–96.6)	(69.9–100.0)	(77.1–95.5)	(70.0–100.0)	(68.6–90.5)	(69.9–100)
	≥500	63	98.0	76.9	96.0	76.9	88.0	84.6	92.0	84.6
		(50/13)	(88.0–99.9)	(46.0–93.8)	(85.1–99.3)	(46.0–93.8)	(75.0–95.0)	(53.7–97.3)	(79.9–97.4)	(53.7–97.3)

*IA+/IA-  =  Treponemal immunoassay reactive/Treponemal immunoassay nonreactive.

Sens  =  sensitivity; Spec  =  specificity; SYD = Sydney (Architect Syphilis Chemiluminescence IA); MEL = Melbourne (Trepanostika TP recombinant Enzyme IA)

Differences between estimates were considered to be statistically significant where 95% CI were not overlapping.

By HIV status, there were no statistically significant differences observed in POC test sensitivities. POC test specificities were lower among HIV positive compared to HIV negative specimens; however the difference was only statistically significant for the Determine test (81.5% vs. 97.8%). Though not statistically significant, POC test specificities were lower among those with CD4 count≥500 cells/mm^3^ compared to <500 cells/mm^3^ across all POC tests.

Table 4 shows sensitivity of syphilis POC tests compared to clinical syphilis stage. Sensitivity of the Determine test was 100% for both primary and secondary syphilis however was lower for each of the other three tests among primary compared to secondary syphilis (none were statistically significant). The Bioline test was however statistically significantly less sensitive compared to the Determine test among primary syphilis cases. No other differences were noted between any of the tests across any other these clinical categories.

**Table 4:** Sensitivity of syphilis POC tests compared to clinical syphilis stage.

		Determine	Onsite	DPP (Trep)	Bioline
Clinical stage	N	Sens	Sens	Sens	Sens
		(95% CI)	(95% CI)	(95% CI)	(95% CI)
Primary	53*	100.0	88.7	84.9	77.3
		(91.6–100.0)	(76.3–95.3)	(71.9–92.8)	(63.5–87.3)
Secondary	70	100.0	98.6	94.3	94.3
		(93.5–100.0)	(91.2–99.9)	(85.3–98.2)	(85.3–98.2)
Early latent	91	95.6	93.4	92.3	86.8
		(88.5–98.6)	(85.7–97.3)	(84.3–96.6)	(77.7–92.7)
Late latent/unknown duration	25	96.0	88.0	84.0	84.0
		(77.7–99.8)	(67.7–96.8)	(63.1–94.7)	(63.1–94.7)
Past/treated	248**	93.1	89.9	88.3	84.7
		(89.1–95.8)	(85.3–93.2)	(83.5–91.9)	(79.4–88.8)

Clinical stage  =  documented in medical records for patient specimens; Sens = sensitivity; *Among Primary syphilis, 2 specimens were IA non-reactive but PCR positive. ** Among past/treated syphilis, 5 specimens were IA non-reactive.

Differences between estimates were considered to be statistically significant where 95% CI were not overlapping.

Table 5 shows POC test sensitivities among IA reactive specimens stratified by RPR titre. POC test sensitivity was significantly higher among high titre (RPR≥8) syphilis infections (range: 94.6–99.5%) than for RPR non-reactive infections (range: 76.3 - 92.9%), with the Determine test demonstrating superior sensitivity to the other three tests across high and low RPR titres and non-reactive RPR specimens. The Determine test sensitivity was statistically significantly higher compared to the Bioline test in the high and low titre categories and compared to all other tests in the non-reactive RPR category.

**Table 5:** POC test sensitivity among reference Treponemal immunoassay reactive specimens, by RPR reactivity and titre.

Reference results			Sensitivity (95% Confidence Intervals)			
IA	RPR	N	Determine	Onsite	DPP (Trep line)	Bioline
R	R:≥8	404	99.5	98.3	95.3	94.6
			(98.0–99.9)	(96.3–99.2)	(92.6–97.1)	(91.7–96.5)
R	R:1,2 or 4	121	97.5	93.4	93.4	85.1
			(92.4–99.4)	(87.0–96.9)	(87.0–96.9)	(77.2–90.7)
R	NR	211	92.9	81.0	77.3	76.3
			(88.3–95.8)	(75.0–86.0)	(70.9–82.6)	(69.9–81.8)

Sensitivity of POC test result compared to reference treponemal immunoassays (IA); RPR = reactive plasma reagin; R = reactive; NR = non-reactive

Differences between estimates were considered to be statistically significant where 95% CI were not overlapping.

Inter-reader variability analysis (discrepancy rate between results recorded by two technicians) revealed a range of discrepancies across the tests: Determine 0.2% (n = 3), Bioline 1.2% (n = 15), DPP 1.5% (n = 18) and Onsite 2.7% (n = 33). Technician's comments indicated that many of these discrepancies occurred when results lines on the tests kits were faint.

Technicians (n = 2) found all the four POC tests were “simple” or “very simple” to conduct. Overall interpretation of the POC result was considered to be “relatively easy” for all POC test devices, however technicians raised concerned regarding interpretation of faint or weak bars. Faint bars were reported to have occurred “sometimes” with all the devices except the Determine, leading to discrepancies in initial results between technician 1 and 2. Technicians noted some problems with the nitrocellulose strip in the Determine test and the cassette casing for the DPP and Onsite devices.

## Discussion

This paper reports results from a large, dual-site, head-to-head evaluation of performance of four commercially available syphilis POC test devices using stored sera. This is the first such study exploring the impact of syphilis stage and HIV co-infection on POC performance. Our results provide evidence supporting the good performance of treponemal tests currently available, with the Determine test demonstrating overall superior performance and with technicians experiencing the least number of challenges in interpretation of results. Uniquely demonstrated in our study, test sensitivities did not differ by HIV status but did differ by clinical stage and RPR titre.

Though much of the current published literature focuses on the use of these POC tests as part of antenatal screening programs in developing countries, there may be a role for these tests, particularly those able to differentiate potentially active from inactive/past infection, in other settings, including developed countries among populations with high rates of syphilis and HIV co-infection. Our results suggest that such a role be considered in the context of ongoing routine serology to identify false negative results, given the possible poorer sensitivity among primary syphilis of some of these tests.

Overall, the Determine test had the highest sensitivity (97.3%), while all four had similarly very high specificities (97.2–98.5%). The overall kappa statistic for each test, a summation of the overall performance of each test against the reference standard, was highest for Determine TP suggesting very substantial agreement between the POC test and laboratory reference results. These individual test performances are not dissimilar to others reported in the literature [12], [15], [16], [19], [26]–[34] and support their use as screening tools for syphilis, particularly in resource-limited settings where routine laboratory-based screening is limited.

We observed no difference in sensitivity by HIV status, however, we did note a trend towards reduced test specificity among HIV positive compared to negative specimens. Determine was the only test showing a statistically significant lower specificity among HIV positive specimens. This phenomenon has been described elsewhere [29], [35] and may be the result of immune activation and subsequent deranged B-cell function in HIV [36] which presumably can lead to false positive tests. This is clearly important in settings such as Europe, USA and Australia where among gay men, up to 60% the syphilis infections are in HIV-positive individuals [37]–[39], such that approximately 1 in 6 reactive tests could be a false positive in this population. We were unable to detect any differences in performance by CD4 count though specificities appeared to be somewhat lower among those with HIV and CD4 counts ≥500 cells/mm^3^. As numbers were small, these results should be interpreted with some caution.

The Determine test was the best performing POC test and was able to identify 100% of specimens in both primary and secondary stage infection. The other three tests appeared to have a lower sensitivity among primary infection, particularly the Bioline test with a sensitivity of only 77.3%. In all of these tests the sensitivity increased in secondary infections but did not reach 100%. These tests would therefore miss a number of primary and secondary infections. In many countries all patients with a genital ulcer are treated for syphilis as identification of early syphilis infections is important to prevent ongoing transmission and is a key STI control strategy.

Although the DPP Screen and Confirm syphilis test has a lower sensitivity compared with the Determine test, it does have the ability to detect both treponemal and non-treponemal antibodies, and therefore help distinguish past, treated infection from possible active infection with some encouraging lab and field results [34], [40]. However in this paper, as we wished to compare performance across tests and investigate clinical and serological factors that might affect performance, we have focused on reporting only the sensitivities and specificities of the treponemal line of the DPP test compared to reference IA tests.

The strength of our study was the majority of specimens tested were collected from clients attending large urban sexual health clinics with substantial expertise in syphilis and HIV and providing for a population at highest risk of syphilis (urban gay men) making our study sample well-suited to evaluate potential differences in POC test sensitivity by clinical stage and HIV status. A smaller proportion of our study population were women and although information regarding pregnancy status was not collected, it is likely that fewer than 1% of these women would have been pregnant in this setting (B.Donovan, personal communication).

There are also a few limitations to consider. The two laboratories providing reference results each used a different treponemal IA. Both are highly sensitive and specific and as relative ranking of POC test performance did not differ by site, we believe the use of two different reference IAs did not bias our results. Our study was conducted in a laboratory environment and the tests were performed by skilled technicians with experience in POC test devices, therefore likely represent a best performance scenario. Though results may differ in a clinical setting, the relative performance of the tests would be expected to remain similar. As these tests are not quantitative, training regarding the interpretation of results required technicians to record a positive result if any band appeared on the test strip at the test line site. It is possible that some faint lines were interpreted by our technicians as positive that may have been called negative by others. Though some evaluations have demonstrated lower test sensitivities using whole blood in field settings [12], [16] compared to sera in a laboratory setting, a recent evaluation has contradicted these findings showing similar performance across specimen type [34].

In conclusion, our results support the potential role for these tests to be implemented more broadly supplementing existing antenatal screening strategies in resource limited settings. Their use in developed countries might also be considered in populations with high rates of syphilis and HIV co-infection and where loss to follow-up is a significant risk. This might be either as part of a screening strategy using the Determine test or to distinguish potentially active from past/treated infection using a newer dual platform test. Field evaluations of selected POC tests are needed to determine the local performance in the hands of end users, relevance, acceptability, economic costs and potential impact associated with implementation in each setting.
